# Cytotoxicity Assessment of Nanoplastics and Plasticizers Exposure in *In Vitro* Lung Cell Culture Systems—A Systematic Review

**DOI:** 10.3390/toxics10070402

**Published:** 2022-07-20

**Authors:** Fabiana Clérigo, Sandra Ferreira, Carina Ladeira, Ana Marques-Ramos, Marina Almeida-Silva, Luís André Mendes

**Affiliations:** 1 H&TRC—Health & Technology Research Center, ESTeSL—Escola Superior de Tecnologia da Saúde, Instituto Politécnico de Lisboa, 1990-096 Lisbon, Portugal; 2018574@alunos.estesl.ipl.pt (F.C.); 2018425@alunos.estesl.ipl.pt (S.F.); carina.ladeira@estesl.ipl.pt (C.L.); ana.ramos@estesl.ipl.pt (A.M.-R.); marina.silva@estesl.ipl.pt (M.A.-S.); 2 Comprehensive Health Research Center (CHRC), Universidade NOVA de Lisboa, 1150-082 Lisbon, Portugal; 3 Centro de Ciências e Tecnologias Nucleares (C2TN), Instituto Superior Técnico, Universidade de Lisboa, Estrada Nacional 10, ao Km 139.7, Bobadela-Loures, 2695-066 Lisbon, Portugal; 4 Animal Ecology Group (GEA), Universidade de Vigo, 36210 Vigo, Spain

**Keywords:** cytotoxicity, *in vitro*, lung cells, nanoplastics, plasticizers

## Abstract

Emerging contaminants such as nanoplastics (NPs), as well as manufacturing by-products such as plasticizers, have gained global attention and concern due to their limited biodegradability and their potential impact on human health, in particular the effects on respiratory tissue. In parallel, *in vitro* cell culture techniques are key to the assessment and characterization of toxic effects and cellular mechanisms in different types of tissues and should provide relevant information to understand the hazardous potential of these emergent contaminants. This systematic review presents the main results on the current knowledge of the effects of NPs and plasticizers on lung cells, as assessed with the use of *in vitro* cell culture techniques. From the selected studies (*n* = 10), following the PRISMA approach, it was observed that cell viability was the most frequently assessed endpoint and that most studies focused on epithelial cells and exposures to polystyrene (PS). It was observed that exposure to NPs or plasticizers induces cytotoxicity in a dose-dependent manner, regardless of the size of the NPs. Furthermore, there is evidence that the characteristics of NPs can affect the toxic response by promoting the association with other organic compounds. As such, further *in vitro* studies focusing on the combination of NPs with plasticizers will be essential for the understanding of mechanisms of NPs toxicity.

## 1. Introduction

Plastics are materials in high demand for several applications due to their physicochemical versatility. These materials are characterized by their chemical stability, exhibiting high durability and firmness, yet may be moldable when heated [1]. These features, along with their low production cost, make plastics highly sought after for every type of use [2].

Due to the massive production of plastics since the 1950s and consumption in everyday life [3], the release to the environment of plastics and substances used in their production and the resulting pollution has been increasing until the present [4]. The impact of these materials is transversal to all ecosystems and their components: plants, animals, and humans [5]; thus, it is important to assess the hazardous consequences [6].

Plastics can be categorized according to their source as primary or secondary [7]. Primary plastics result from intentional manufacturing [8]. Secondary plastics are those that result from the degradation of larger plastic fragments by photodegradation, oxidation, microbial degradation, hydrolytic degradation, and/or mechanical disintegration [9].

Over time, plastics can be fragmented into microplastics (MPs, plastics with diameter <5 mm) [2] and/or nanoplastics (NPs), which can be considered lower than 1000 nm or 100 nm, according to the source literature [8]. In order to better understand the effects of the exposure to NPs, the criteria applied has a range of up to 1000 nm.

The limited biodegradable nature of these polymers [10] presents an environmental impact that has gained global attention and concern, especially due to the effects on health by the exposure of MPs and NPs and their compounds [11,12,13].

MPs have been detected in processed foods, beer, seafood, and sugary drinks, among others. In addition to the digestive route, MPs/NPs can be inhaled and come into direct contact with the entire respiratory tract: the mucus layer, the pericyte layer, the ciliated and non-ciliated secretory cells, and the basal cells [14,15] Within the lungs, the epithelial cells covering the airway surfaces and alveoli are the first target cells for exposure to inhaled substances [16], with plastic particles causing lung inflammation and genotoxicity by chronic exposure [14]. There is also evidence from *in vitro* studies with lung epithelial cells that NPs may have adverse health effects by inducing oxidative stress and inflammatory responses, followed by cell death and epithelial barrier destruction, resulting in tissue damage and lung disease after chronic exposure [17]. Thus, MPs and NPs are considered of high relevance in the context of public health [18], as well as the exposure to related contaminants, such as plasticizers [19,20].

Plasticizers, namely phthalates, are also present in most everyday items, such as toys, packaging, and medical devices [21,22]. Their release and increased availability in the environment across different ecosystems are a result of fragmentation and degradation of plastics [23]. The long-term consequences of exposure to plasticizers have also been demonstrated, including their effect on human health; for example, at the level of the endocrine and reproductive systems [24,25]].

*In vitro* models of human cells have allowed a better understanding of the cellular mechanisms, including those related to contaminant exposure [26] and the onset of diseases [16]. Both single cell type and co-culture systems have shown to be fundamental to the study of cell–cell interaction and molecular level analysis, providing valuable information in terms of the mechanisms behind cell cycle, proliferation, and cell death, such as senescence, apoptosis, and necrosis, in various scenarios [27,28,29]. Their use in the study of NPs and plasticizers should provide important advances in scenarios closer to reality in terms of responses and effects of exposure.

This review of the literature aims to summarize the current evidence of NPs and plasticizers *in vitro* exposure in single or co-culture systems, using human and other animals’ lung tissue. This should provide relevant information on the exposure conditions that trigger responses in lung cells in single and co-culture systems. This will contribute towards the development of experimental designs looking to assess the level of deregulation, toxicity, and the associated mechanisms in cells from the respiratory tract, induced by NP and plasticizer exposure. A population, exposure, and outcome (PEO) statement was developed by the team as an aid to identify search terms and inclusion/exclusion criteria as appreciated for addressing this systematic review (Table 1).

## 2. Materials and Methods

The systematic review was conducted using the general principles of the PRISMA (preferred reporting items for systematic reviews and meta-analyses) [30], that aimed to harmonise the development and reporting of systematic review protocols.

### 2.1. Search Strategy

For the literature search, no limits were set on study designs, language, or year of publication. The authors searched three electronic databases: PubMed, Web of Science, and Scopus, from their inception until February 2022 for studies that assessed the *in vitro* response of lung cells to MPs, NPs, or plasticizers. The search strategy was defined using combined terms related to the studied contaminants (“microplastics”, “nanoplastics”, or “plasticizers) in combination with those related to culture techniques (“*in vitro* techniques”, “cell culture techniques”, or “co-culture techniques”), the key targets (“respiratory system”, or “lung” and “epithelium”), and cellular response to the contaminants (“cellular events”, “cell deregulation”, or “toxicity”). Additionally, publications citing the selected articles were checked, as well as further searches in the Google Scholar engine database were performed to retrieve additional articles, the latter identified as grey literature.

### 2.2. Screening and Eligibility Criteria

The Rayyan software was used during the screening phase. Three authors (F.C., S.F., and L.A.M) first screened all abstracts and titles resulting from the search to eliminate clearly irrelevant studies. The same authors then screened full-text articles and made final eligibility decisions based on inclusion/exclusion criteria, as follows. For inclusion, studies were shown to have an experimental *in vitro* design, in which effects of MPs, NPs, or exposure to plasticizers to *in vitro* lung cells were assessed using different endpoints at the cellular and molecular level. Excluded were systematic reviews, cohorts, cross-sectional and *in vivo* studies, comments, consensus reports, editorials, guidelines, and protocols. In all phases, disagreements were resolved by discussion and consensus between four of the authors (F.C., S.F., L.A.M, and M.A.-S.).

### 2.3. Study Selection

Firstly, the abstracts of the retrieved references were read, and those selected included any of the conditions specified (i.e., microplastics, nanoplastics, plasticizers, respiratory system, lung epithelial cells, single-cell culture, and co-culture techniques), as well as cellular events, such as cell deregulation, cytotoxicity, or DNA damage, and exposure in multicellular scenarios. Secondly, the full paper of the selected abstracts was read to confirm if it fulfilled all selection criteria. Disagreements were resolved by consensus and in consultation with a fourth author (M.A.-S).

For the purpose of this study, the criteria for NPs’ size were considered as ≤1000 nm [8].

### 2.4. Data Extraction

Extracted data from each article was registered in a predesigned Excel form regarding organism, tissue and cell type, culture technique, contaminant type and size, concentration range, exposure duration, outcome, and any other relevant conclusion.

### 2.5. Synthesis of the Evidence

The diversity in study designs, models, doses, and ways of assessing exposure and outcomes did not allow us to carry out a comparative quantitative analysis. Instead, the authors provided a qualitative overview of the extracted data and characterized the studies, exposures, outcomes, and main findings.

## 3. Results

**Figure 1:**
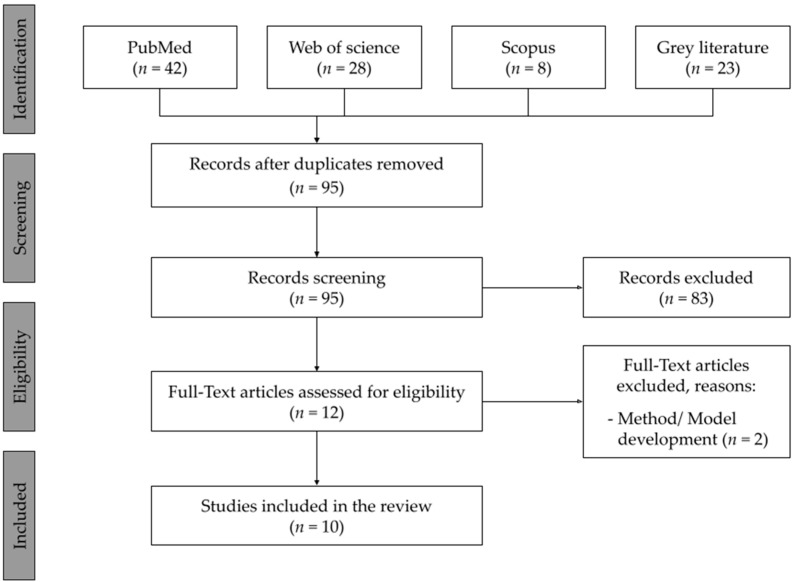
Summary of the inclusion and screening of articles following the PRISMA approach [30].

The full PRISMA search strategy is provided in Figure 1. The primary phase of the search on the PubMed, Web of Science and Scopus databases and grey literature returned 101 studies, from which 95 abstracts were screened after removing the duplicates, and 12 full texts were considered eligible. After a deeper assessment of the full papers, a total of 10 were included for this review and 2 were excluded for the type of study (method/model development, not in accordance with the study of cytotoxic effects), narrowing down the number of included papers to 10.

The main data from the studies selected by the PRISMA approach are displayed in Table 2.

All the selected studies were performed in single culture system (10 out of 10), with none focusing on co-culture systems.

All selected studies used human cells (10 out of 10), with the majority being A549 alveolar epithelial cells (7). BEAS-2B bronchial cells only (1) and WI38va13 cells (1), in addition to a comparison between BEAS-2B and HPAEpiC alveolar epithelial cells (1) were also included.

The exposure contaminants in the selected papers were NPs (6), plasticizers (2) and a combination of NPs and plasticizers (2), as shown in Table 2. As for the contaminants to which cells were exposed to, polystyrene (PS) NPs are the only ones studied for lung cells, whereas the most common tested plasticizer was DEHP.

In regard to concentrations, the maximum concentration tested was 300 µg/mL [31], with the majority of assessment performed in a range up to 100 µg/mL. For the plasticizers, their concentrations varied from 1–100 μM for DEHP and for MEHP [36] and 0.03–20 ng/cm^2^ for DBP [37].

**Figure 2:**
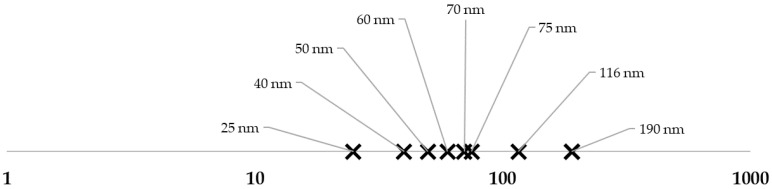
NPs’ sizes (nm) from the studies included for review: 25 and 70 nm [31]; 40 nm [17]; 50 nm [32]; 60 to 75 nm [35]; 116 nm [20]; 190 nm [33,34] Upper limit of 1000 nm defined based on reviewed literature [8].

As for the NPs used in these studies, the sizes ranged from 25 up to 190 nm, as shown in Figure 2, with the majority between 10 and 100 nm.

The main findings of these studies were clustered according to the type of endpoints assessed: cell viability, type of cell death, DNA damage, and protein expression. The most frequently measured endpoint was the decrease in cell viability (8 of 10) in A459, BEAS-2B, and WI38va13, followed by apoptosis or cell death (7 of 10) in A459, BEAS-2B, and HPAEpiC.

The substantial majority of studies presented effects in cell viability due to NPs or plasticizer exposure, albeit at different concentrations. A decrease in cell viability in a dose-dependent manner for both contaminants was mainly observed (5), but also related to NP size (2). Furthermore, surface charge-dependent internalization for PS NPs exposure was reported (1). However, in other studies (2), no relation between cell viability and dose, size, charge, or time was observed (2).

The observed effects on cell viability were organized according to the contaminant type, the cell line used, the corresponding exposure period, and the concentration range in Table 3. According to the data from the studies, exposure to concentrations of NPs equal to or greater than 10 μg/mL for 24 h resulted in a significant effect on cell viability in A549 and BEAS-2B. For the HPAEpiC cell line, the concentration from which significant effects are observed on cell viability are 15 μg/mL [17].

Regarding the plasticizer data, a 6 h exposure to MEHP from 5 µM resulted in a significant decrease in viability, with values below 70% of the control, whilst significant effects were also observed at 24 and 48 h and at concentrations above 10 µM. As for DEHP, significant effects after 24 and 48 h are seen in concentrations of 100 µM and above [36]. Simultaneous exposure to NPs and plasticizer shows significant effects on cell viability for NPs at concentrations above 200 μg/mL and for plasticizers they show effects at the concentration used of 5 μg/mL [20].

Regarding the effects of NPs and plasticizers on apoptotic pathways, the expression of pro-apoptotic proteins along with the activity of the enzymes caspase-3, caspase-8, caspase-9, BAX, and BCL2 were studied for PS NPs exposure at concentrations between 0 and 300 µg/mL, considering particles of 25 nm and 70 nm of diameter, and 0 and 30 µg/cm^2^, for particles with 40 nm of diameter.

The inhibition of cell viability was reportedly associated with oxidative stress, inflammatory response, and endoplasmic reticulum stress caused by misfolded protein aggregation, which varied according to the contaminant cellular uptake. Other effects, such as perturbance in the energy metabolism, changes and a loss of mitochondrial membrane potential, and an increase in autophagic activity were observed.

## 4. Discussion

One of the emerging environmental issues and threats is plastic pollution, due to the hazard for living species related to the additives and adsorbed chemicals present in plastic [39,40]. Considering the abundant presence and contamination of MPs and NPs in the indoor and outdoor environments, namely in soil, water and the atmosphere, there is a need to understand the degradation processes and fate of these particles in the environment, as well as their effects for human health [41].

Phthalates are found in different matrixes: soil, sediment [42], biota [43], wastewater [44], and outdoor and indoor air [45], and are reported with high concentrations in airborne particles and settled dust for indoor air. Their presence and abundance contribute to potential human exposure. From all exposure pathways, inhalation represents a major mode for the phthalate entrance, which is associated with compound volatility [46]. The temperature and particle mass concentration highly contribute to short-term changes in its airborne concentration [46].

The respiratory system is the entrance for inhaled particles, turning it in a target for the toxicity of nanoparticles [47]. Due to the permeability of the alveolar epithelium, there is a risk of adverse effects according to the type and concentration of the inhaled particles [48].

In this review, all studies included were focused on the exposure of epithelial cell lines, namely A549, followed by BEAS-2B cells. This is understandable given their thorough use of both cell types in studies that associate lungs and toxicity [49]. However, the lack of studies on lung fibroblasts, e.g., IMR90, shows that the impact of NPs and plasticizers in the lung connective tissue may be downplayed, as well as the role that this tissue can have in the malignization of epithelial cells, namely through the development of senescent phenotypes [29].

Given the advantages of using co-culture systems [50], which allow the study of the phenotype of different cell types simultaneously, mimicking the actual tissue, the authors expected that the use of this methodology in the study of exposure and co-exposure of NPs and plasticizers in lung cells would have resulted in several publications. However, this systematic review showed that all studies were carried out in single cell lines, as such little is known in terms of NPs and plasticizer toxicity in complex realistic multicellular exposure scenarios and outcomes. The use of more complex systems, such as co-culture, would allow a better understanding of the interactions between different cell types in response to NPs and related contaminants.

The most frequent effects identified in the various studies are related to the effect of contaminants on cell viability/cytotoxicity. Other endpoints such as oxidative stress, and gene and protein expression have been observed occasionally. These endpoints have been frequently used to observe toxicity at sub-lethal concentrations of other contaminants [35,51], as well as NPs in other cell types, e.g., gastric tissue [52]. Cell viability may be significantly affected or not, depending on the contaminant concentration and co-exposed contaminants. When the NPs exposure was considered exclusively significant effects at low and moderate concentrations were observed; however, at concentrations >100 µg/mL, the evidence was not consistent. As such, a larger number of studies in this regard are required to understand the different levels of effects prior to cytotoxicity mechanisms in respiratory tissue.

Compared to MPs and macroplastics (MAPs), NPs present different physicochemical and biological properties; however, their environmental measurements are hardly available [53]. It is known that the size of the particle strongly influences cell–particle interaction [54]; however, there is a lack of information regarding the individual role of their size, surface chemistry, and shape in the cellular internalization.

Other studies, such as from Wahl et al. [55], demonstrated the presence of NPs with a size ranging from 20 to 150 nm are covering three of the most common plastic families: polyethylene, polystyrene, and polyvinyl chloride. Furthermore, this study provides evidence that NPs production by plastic degradation can occur in the soil matrix.

This review showed that the type of plastic most used as a contaminant is PS. Considering the wide diversity of plastics existing in the environment at present and the calls for an “ecology of plastics” [56], it should be assumed that this is still a wide-open field for research, with the need for further studies with different polymer types. Furthermore, the NPs presented in this study ranged between 25 and 190 nm in size. As the NPs definition used in this study (<1000 nm) covers a large range of sizes, there is still a large knowledge gap regarding NP-related effects. However, there is evidence that sub-cellular responses (DNA damage, and protein expression) are associated with NPs internalization, which hardly occurs for particles with sizes above 500 nm [57].

In addition to evidence of a dose-mediated response to NPs on cell viability [31], changes in shape and surface modifications were shown to also affect the NPs internalization by cells [32]. Studies with a surface modification of nano-sized materials have shown to be determinant in the toxicity of these types of materials at both sub-cellular and individual level [58]. In a study by Petithory et al. [54], it is highlighted that the occurrence of a delay in internalization increased with adsorbed nanoparticle size, characterizing this internalization by a minimal threshold that corresponds to 35 nm nanoparticles that are not internalized during the 12 h incubation. Conversely, Liu et al. have shown that 50 and 500 µm sized plastics can be passively internalized [57]; as such, further studies should take into consideration not only the concentration but also the shape and size of the NPs studied in lung cells and the incubation time to define threshold levels.

Also considering the modification of PS, this may alter their biological activity, by promoting the combination with other contaminants, namely organic compounds, which can change the environmental transformation and biotoxicity of those [59]. For organic compounds such as benzo(a)pyrene (BaP), which its presence in the air is related to the attachment to the airborne solid particulates [60], its adsorption can affect physicochemical properties, and consequently, the cellular response to NPs [61,62]. According to the articles included in this review [20,33,34], there is evidence of alterations in cell viability with exposure to phthalates and BaP nanoclusters.

The study of apoptotic markers in lung cells exposed to NPs and plasticizers verified the disturbance of the mitochondrial membrane potential [31,33]. It was also observed that the alteration ATP production and deregulation of the energy production mechanism [33,35] contributed towards cell damage [17,20,37]. Consequently, cell death has been reported [17,49] particularly for long exposure periods [17]. These findings were also reported *in vivo* studies [63]. The effects evidenced in these studies at energy production mechanism show how NPs can have a negative impact and be a possible stressor on the energy metabolism and efficiency. This means that the mitochondria and other organelles have a role in the toxicity of NPs, as stated by other authors [63,64].

The NPs can sorb phthalate esters and affect their toxicity, highlighting the importance of studying more realistic scenarios, since NPs may be a carrier to other contaminants, and by particle agglomeration, decrease their uptake. Based on some studies reviewed, it is possible to identify that NPs alone may not have a very significant toxic effect, as evidenced by Shi et al. [20]; however, when combined with other products such as BaP or phthalates the toxic effect is more evident [33,34]. It is important not only to study the effect, but also the interaction between the compounds or complexes created, since it has been shown that PS can increase the cytotoxicity of BaP, which highlights the need for studies on combined contaminants, even if those are not related by the degradation process, and nanoparticles can adsorb other organic compounds. This is a relevant issue as evidence of combination of NPs and other contaminants produce toxic effects to living organisms that were not observed in individual exposure [65].

Finally, due to the inert characteristics of NPs and their low solubility, the methodology used for incubation can also influence the results obtained. This was evidenced in a study by Stock et al. [66] through the use of an inverted cell culture for HepG2 cells, which showed cytotoxicity effects when compared with the non-inverted cell cultures, where no cytotoxicity occurred. This highlights the importance to adapt cell culture conditions to more realistic exposure conditions, including exposure interface, type and size of NPs, and the existence of other contaminants and neighboring tissues.

## 5. Conclusions

This review showed that there are some studies evaluating exposure to NPs or plasticizers using single-cell culture systems and the most used cell line being A549. Whereas individual exposure to these contaminants has been studied, with effects mostly observed on cell viability, there is an absence of more complex culture systems, such as co-culture system studies, which assess the role of other tissues on cell deregulation and toxic effects. Furthermore, the simultaneous exposure of these contaminants cannot be neglected, as there is already evidence of effects due to NPs and organic compound co-contamination.

NPs exposure have an effect in the energy-regulation mechanisms, and it was shown to have the capacity to carry other compounds; however, there is evidence that being a carrier also interferers with the contaminants’ cellular uptake. It is imperative to further evaluate the co-exposure to NPs and plasticizers to lung cells in complex scenarios in the future to better understand the cytotoxicity effects of simultaneous exposure.

## Figures and Tables

**Table 1:** PEO criteria for inclusion and exclusion in the systematic review.

PEO	Inclusion Criteria	Exclusion Criteria
Population	*In vitro* respiratory system cells	Cohorts, cross-sectional and *in vivo* studies, *in vitro* studies on other cell systems
Exposure	Nanoplastics (up to 1000 nm in diameter), plasticizers	Microplastics (>1000 nm)
Outcome	Cell viability, cell morphology, cell fate, DNA damage and protein expression	

**Table 2:** List of papers reviewed clustered according to exposure type and the main findings.

Exposure			Main Findings				References
Contaminant Type		Cell Type	Cell Viability	Cell Death	DNA Damage	Protein Expression	
NPs	PS	A549	✔	✔	✔	✔	[31]
			✔	✔		✔	[32]
			✔				[33]
				✔			[34]
		BEAS-2B	✔	✔		✔	[35]
			✔	✔			[17]
		HPAEpiC	✔	✔			[17]
Plasticizers	MEHP, DEHP	A549	✔	✔		✔	[36]
	DBP	A549			✔	✔	[37]
NPs + Plasticizers	PS + DEHP, DBP	A549	✔				[20]
	PVC + DEHP, DIOP	WI38va13	✔				[38]

DBP—Dibutyl phthalate; DEHP—Di(2-ethylhexyl)phthalate; DIOP—2,3-O-isopropylidene-2,3-dihydroxy-1,4-bis (diphenylphosphino)butane; MEHP—Mono-2-ethylhexyl phthalate; PS—Polystyrene; PVC—Polyvinyl chloride.

**Table 3:** Cell viability effects by concentration range and contaminant (NPs, plasticizers or both).

Contaminant Type	Cell Type	Exposure Time	Concentration Range	Cell Viability	Reference
NPs	A549	24 h	2.5–30 μg/mL	No significant changes at <5 μg/mL Significantly increase at 10 and 15 μg/mL Significant toxic effects at >25 μg/mL	[31]
			10–300 μg/mL	Significant effects at >160 μg/mL	[31]
			500 μg/mL	No significant effects	[33,34]
	BEAS-2B	24 h	0–100 μg/mL	Significantly inhibition at >10 μg/mL	[35]
			0–40 μg/cm^2^	Significantly inhibition at >10 μg/cm^2^	[17]
	HPAEpiC	24 h	0–40 μg/cm^2^	Significantly inhibition at >15μg/cm^2^	[17]
Plasticizers	A549	4 h–24 h	0–1000 µM MEHP	70% reduction at 100 and 1000 µM for MEHP and significant difference at 10–1000 μM	[36]
		24 h–48 h	0–1000 µM MEHP 0–1000 µM DEHP	Significant difference at 5–1000 μM for MEHP 70% reduction at 50 μM for DEHP and a significant difference with 100–1000 μM	[36]
			0–20 ng/cm^2^ DBP	No significant changes observed	[37]
NPs + Plasticizers	A549	24 h	0–1000 µg/mL NPs + 5 µg/mL Plasticizers	Significant effects at >200 μg/mL for NPs and at 5 μg/mL for plasticizers	[20]

## Data Availability

Not applicable.
